# Gastrointestinal symptoms have a minor impact on autism spectrum disorder and associations with gut microbiota and short-chain fatty acids

**DOI:** 10.3389/fmicb.2022.1000419

**Published:** 2022-10-07

**Authors:** Wenlin Deng, Siqi Wang, Fang Li, Fang Wang, Yi Pei Xing, Yongchun Li, Ying Lv, Haoran Ke, Zitong Li, Pin Jing Lv, Hu Hao, Ye Chen, Xin Xiao

**Affiliations:** ^1^Department of Pediatrics, The Sixth Affiliated Hospital, Sun Yat-sen University, Guangzhou, China; ^2^Nanfang Hospital, Southern Medical University, Guangzhou, China; ^3^Department of Gastroenterology, Gastroenterology Endoscopy Center, Hainan General Hospital, Hainan Affiliated Hospital of Hainan Medical University, Haikou, China; ^4^Third Affiliated Hospital of Sun Yat-sen University, Guangzhou, China; ^5^Shenzhen Hospital, Southern Medical University, Shenzhen, China

**Keywords:** autism spectrum disorder, microbiota, short-chain fatty acids, gastrointestinal symptoms, eating behaviors

## Abstract

Children with autism spectrum disorder (ASD) experience gastrointestinal (GI) issues more frequently and severely than children who are typically developing (TD). The connections between gastrointestinal problems, microbiota, and short-chain fatty acids (SCFAs) in ASD are still being debated. We enrolled 90 children, 45 of whom were diagnosed with ASD, and examined the impact of GI disorders on ASD. The six-item GI Severity Index questionnaire was used to evaluate gastrointestinal symptoms, while the Social Responsiveness Scale was used to evaluate autism symptoms. Further, the Children’s Sleep Habits Questionnaire and the Children’s Eating Behavior Questionnaire are used to assess sleep and eating disorders in children. We assessed fecal microbiota by 16S rRNA gene sequencing, and SCFA concentrations by gas chromatography/mass spectrometry. The results revealed that children with ASD exhibited a high rate of gastrointestinal issues (78%), as well as higher rates of social impairment and poor sleeping habits, compared to TD children. However, GI disturbances have a minor impact on autism. In addition, the levels of propionic acid, butyric acid, and valeric acid were significantly higher in the ASD group. Besides, the ASD, TD, and GI subgroups possessed distinct microbiome profiles. These findings suggest that gastrointestinal disturbances have no discernible effect on the core symptoms of autism. Although autism may not cause an increase in GI symptoms directly, alterations in metabolites, such as SCFAs, may cause GI symptoms.

## Introduction

Autism spectrum disorder (ASD) is a heterogeneous neurodevelopmental disorder characterized by impaired social communication and restricted repetitive behaviors (American Psychiatric Association, 2013). The association between gut microbiota and autism spectrum disorder (ASD) has attracted great interest in recent years. Changes in the composition of the gut microbiota and its metabolites are frequently observed in humans and animal models of ASD (Adams et al., 2011; Nankova et al., 2014). Furthermore, probiotics, prebiotics, fecal transplantation, and dietary therapy can change the gut microbiota and influence the progression of neuropsychiatric disorders, including depression, ASD, and anxiety disorders, a manifestation of the so-called gut-brain-microbiota axis (Garcia-Gutierrez et al., 2020; Socała et al., 2021).

Children with ASD who have untreated gastrointestinal (GI) distress have been related to a variety of problems, including sleep, feeding, behavioral, and psychiatric disorders. Previous studies have also shown that GI symptoms are four times more common in children with ASD than in typically developing (TD) children. According to a meta-analysis, individuals with ASD and GI symptoms may exhibit more severe symptoms such as anxiety, self-injury, and aggression (Fattorusso et al., 2019). Numerous publications have hypothesized that the GI issues seen in individuals with ASD are due to an altered gut microbiota composition and have posited a link between disturbed gut microbiota and autism (Fattorusso et al., 2019; Settanni et al., 2021). However, a recent large autism stool metagenomics study discovered only a few direct associations with autism and suggested a model in which the genetic and phenotypic measures of ASD promote a less diverse diet, thereby reducing microbiome diversity (Yap et al., 2021). These studies have reported mixed results regarding GI disorders and bacterial community differences.

Short chain fatty acids (SCFAs) are crucial intestinal microbial byproducts that are also related to microbiota-gut-brain axis function. Propionic acid may have induced autism-like neurobehaviors, including increased aggression, reduced exploratory activity, and isolative and passive behaviors (Choi et al., 2020). low levels of butyric acid, on the other hand, actively modulate neurotransmitter gene expression and alleviate behavioral abnormalities (Rose et al., 2018; Chen et al., 2021). However, the precise relationships among SCFAs, the gut microbiota, and autism are currently unknown.

GI symptoms, the gut microbiome, and its metabolites have been often related to symptoms appearing concurrently with ASD (Miike et al., 2020). For example, 40%–80% of individuals with ASD experience sleep difficulties, which may exacerbate or imitate autistic behaviors (van der Heijden et al., 2018). The most common sleep issues are sleep-onset difficulties, longer sleep latency, earlier wake-up time, and more night awakenings (Cortesi et al., 2010). Food preferences and dietary patterns are believed to be important factors that influence the development of ASD. Nearly 90% of children with ASD show some form of food issues. Picky eating, food refusal, and food selectivity are all prevalent problematic eating behaviors in children with ASD (Kodak and Piazza, 2008).

Therefore, the purpose of this study was to investigate the prevalence of GI symptoms in individuals with ASD and to characterize the influence of the microbiota and GI problems on ASD phenotypes, to further illustrate the cause-and-effect relationships between the two conditions.

## Materials and methods

### Participants

The Southern Medical University Ethics Committee approved the study (NFEC-2020-176), and the parent or legal guardian of each participant provided informed consent. Ninety children were enrolled in the study, the ASD group included 39 boys and 6 girls (mean ± SD age, 5.95 ± 2.36 years), while the TD group included 21 boys and 24 girls (age, 6.13 ± 0.90 years) were enrolled from a local kindergarten (See Table 1). ASD diagnosis was based on the Diagnostic and Statistical Manual of Mental Disorders, 5th edition. The Social Responsiveness Scale (SRS) was used to evaluate social impairment in ASD patients. Physical, neurological, and behavioral tests were performed for each participant. Children who had previously been diagnosed with genetic conditions (such as tuberous sclerosis, fragile X syndrome, and Rett syndrome), had received antibiotics or probiotics within 1 month, or were suffering from trauma, tumors, or other serious nervous system diseases were excluded from the study.

**Table 1:** Summary of subject demographics and characteristics.

	ASD (*n* = 45)	TD (*n* = 45)	Value of *p*
Age (mean ± sd)	5.95 ± 2.36	6.13 ± 0.90	0.6434
**Gender, *n* (%)**			
Male	39(87)	21(47)	<0.001
Female	6(13)	24(53)	
GI disorders *n* (%)	35/45(78)	21/45(47)	0.002
Bristol scores	3.909 ± 1.074	3.523 ± 1.045	0.0909
Height (cm)	104.70 ± 15.86	110.90 ± 7.22	0.0378
Weight (kg)	17.84 ± 5.42	19.12 ± 4.51	0.2682
BMI (kg/m^2^)	16.20 ± 3.57	15.62 ± 3.44	0.4748
Birth weight (kg)	3.11 ± 0.52	3.12 ± 0.53	0.9055
Gestational age (weeks)	38.82 ± 1.33	38.83 ± 2.13	0.9692
**Delivery model *n* (%)**			
Vaginal	20(44)	21(49)	0.68
Cesarean	25(56)	22(51)	
Maternal age (years)	31.69 ± 7.15	30.45 ± 4.88	0.3523
Pre-pregnancy weight (kg)	54.29 ± 7.38	56.09 ± 8.85	0.3344
Maternal weight gain (kg)	12.74 ± 4.50	12.89 ± 3.91	0.8834
Mother’s height (cm)	158.90 ± 5.04	160.00 ± 4.381	0.2973
Mother’s weight (kg)	53.59 ± 8.17	55.01 ± 8.747	0.443
Maternal BMI	21.74 ± 3.05	21.49 ± 3.70	0.7407
Neonatal complications, *n*(%)	30/44(68)	19/33(58)	0.338
**Use of calcium supplement, *n***			
Yes	38	21	0.002
No	4	14	
**Use of Folic acid supplement, *n***			
Yes	41	34	1
No	1	1	
Pregnancy complications, *n* (%)	4/39(10)	6/42(14)	0.831
Maternal smoking	0	0	
Maternal drinking history	0	0	
**Maternal education level, *n* (%)**			
Secondary or less	10/43(23)	10/42(24)	0.952
University	33/43(77)	32/42(76)	
Paternal age	38.40 ± 6.46	38.17 ± 6.54	0.8705
Paternal height	171.70 ± 4.85	171.70 ± 4.37	0.9714
Paternal weight	72.36 ± 18.35	70.74 ± 10.27	0.6233
Paternal BMI	24.52 ± 6.00	24.03 ± 3.62	0.6529
Paternal smoking	20/44(45)	11/44(25)	0.045
Paternal drinking history	10/44 (23)	2/44(5)	0.013
**Paternal education level, *n* (%)**			
Secondary or less	11/43 (26)	8 /42(19)	0.47
University	32/43 (74)	34/42(81)	

GI, gastrointestinal symptoms; TD, typical development.

### Questionnaire analyses

The SRS includes 65 items that assess social perception, cognition, communication, motivation, and autistic behavior. Each category is scored on a scale of 1 to 4, with higher scores indicating greater social impairment (Chan et al., 2017). The Children Sleep Habits Questionnaire (CSHQ) includes 41 questions, 33 of which are scoring items, and evaluates eight aspects (bedtime resistance, sleep-onset delay, sleep length, sleep anxiety, night waking, parasomnia, sleep disordered breathing, and daytime sleepiness). Items are scored on a three-point scale: “usually” indicates a sleep behavior that occurs 5 to 7 times per week (3 points), “occasionally” indicates a behavior that occurs 2 to 4 times per week (2 points), and “rarely” is used to refer to behaviors that occur 0–1 times per week (1 point). The more severe the sleep disorder, the higher the total score. The criterion for evaluating sleep disorders was a total CSHQ score of 41 (Li et al., 2009).

The Children’s Eating Behavior Questionnaire (CEBQ) consists of 35 items divided into eight subscales, each with 3 to 6 items. Parents were asked to rate their child’s eating habits on a 5-point Likert scale (never, rarely, sometimes, often, always; 1–5). GI symptoms were assessed using a 6-item Gastrointestinal Severity Index (6-GSI), which assessed constipation, diarrhea, stool consistency, stool smell, flatulence, and abdominal pain. The Bristol stool scale is a visually graded stool density scale. Types 1–2 indicate constipation; types 3–4 are optimum stools because they are easier to pass; and types 5–7 indicate diarrhea and urgency.

### Microbiome

Fecal samples were collected at home in the morning or on the night before and stored at −18°C. The specimens were transported in coolers with ice packs to the research facility within 6 h and then frozen at-80°C until DNA extraction (Deng et al., 2022). The cetyl trimethyl ammonium bromide/sodium dodecyl sulfate method was used to extract total genomic DNA from samples. The DNA concentration and purity were monitored on 1% agarose gels. The DNA was diluted to 1 ng/l in sterile water according to its concentration. 16S rRNA/18SrRNA/internal transcribed spacer (ITS) genes of distinct regions (16SV4/16SV3/16SV3-V4/16SV4-V5, 18S V4/18S V9, ITS1/ITS2, and Arc V4) were amplified using specific primers (for example, 16S V4: 515F–806R, 18S V4: 528F–706R, 18S V9: 1380F–1510R) with the relevant barcodes. All PCR procedures were carried out using Phusion® High-Fidelity PCR Master Mix (New England Biolabs, Ipswich, MA, United States). The PCR products were mixed in equidense ratios, and the mixture of PCR products was purified using a Qiagen Gel Extraction Kit (Qiagen, Hilden, Germany). Sequencing libraries were generated using the TruSeq® DNA PCR-Free Sample Preparation Kit (Illumina, San Diego, CA, United States) in accordance with the manufacturer’s recommendations, and index codes were added. Library quality was assessed on a Qubit 2.0 Fluorometer (Thermo Fisher Scientific, Waltham, MA, United States) and the Bioanalyzer 2100 system (Agilent Technologies, Santa Clara, CA, United States). Finally, the library was sequenced on an HiSeq2500 platform (Illumina) and 250-bp paired-end reads were generated.

### Short-chain fatty acids

As reported previously (Wang et al., 2019), gas chromatography–mass spectrometry (GC–MS; Santa Clara, CA, United States) was used to separate SCFAs, including acetic, propionic, butyric, and valeric acids. The frozen stool samples (500 mg) were thawed on ice before being accurately weighed into 10-ml centrifuge tubes. Then, 5 ml of ultrapure water was added to the mixture, which was shaken for 5 min. The mixture then underwent ultrasonication at room temperature for 10 min before being centrifuged at 13,000 rpm at 4°C for 10 min. Then, 1 ml of the supernatant was added to a 10-ml centrifuge tube with 50 μl of 2-ethylbutyric acid as an internal standard; the tube was shaken and mixed for 1 min, allowed to stand for 20 min, treated with 10 μl of 50% sulfuric acid and 0.5 g of anhydrous sodium sulfate, shaken and mixed for 1 min for acidification and salting out, and then treated with 2 ml of anhydrous diethyl ether. The supernatant was shaken for 5 min before being centrifuged at 6,000 rpm at 4°C for 5 min and analyzed using GC–MS.

### Analysis

Microbiota statistics displayed with R, QIIME software (for details, see Supplementary File 1), and GraphPad Prism (GraphPad Software Inc., San Diego, CA, United States) were used to analyze the data. All data were described using means, standard deviations (SDs), and percentages. Two-tailed Student’s *t*-test and chi-squared test were used to compare the levels between the two groups. Spearman’s correlation analysis was used to examine the links among microbiota, SCFA, and scores for each scale. The value of *p* was assumed to be significant at *p* < 0.05.

## Results

### Participant characteristics

From October 2020 to December 2021, 90 children, including 45 children with ASD and 45 TD children. As shown in Table 1, the number of boys was significantly higher than the number of girls in the ASD group. The incidence of gastrointestinal symptoms in the ASD group was 78% (35 cases), while it was 47% (21 cases) in the TD group, and the difference between the groups was statistically significant. The most common GI symptoms in the ASD group were smelly stools and constipation. The findings also showed significant differences in height, calcium use among the mothers, and in cigarette smoking and alcohol consumption among the fathers. No significant intergroup differences were observed in Bristol score, parents’ BMI, birth weight, gestational age, delivery model, neonatal complications, folic acid supplementation, and maternal education (χ^2^-test and Wilcoxon rank sum test).

### Sociality, eating, and sleep patterns in children with ASD and TD children

We hypothesized that ASD would have a negative effect on sociality, eating, and sleep behaviors. As expected, in comparison with the TD group, the ASD group showed significantly higher SRS and CSHQ scores (including higher scores for bedtime resistance, sleep duration, parasomnias, daytime sleepiness, and sleep onset delay subscales; see Supplementary Table S1). While the two groups showed no statistically significant differences in the children’s eating behavior questionnaire scores (Table 2; *p* < 0.001), food fussiness and desire to drink subscale scores were notably higher in the ASD group (Supplementary Table S2). On the other hand, as Table 3 demonstrates, GI symptoms significantly affected only the CSHQ scores rather than the SRS and CEBQ scores.

**Table 2:** Scored on eating, sleeping, and sociability.

	ASD	TD	Value of *p*
SRS	99.71 ± 24.66	40.23 ± 17.51	<0.0001
CEBQ	98.58 ± 16.77	97.66 ± 11.75	0.7659
CSHQ	57.78 ± 6.56	51.48 ± 10.19	0.0008

SRS, Social Responsiveness Scales; CEBQ, Children’s Eating Behavior Questionnaire; CSHQ, Children’s Sleep Habits Questionnaire; TD, typical development.

**Table 3:** Gastrointestinal symptoms affected on sociality, eating and sleep.

	ASD^GI^	ASD^NOGI^	TD^GI^	TD^NOGI^
Subjects (*n*)	35	10	22	23
SRS	98.06 ± 23.85	105.00 ± 27.75	41.62 ± 12.20	38.91 ± 21.63
CEBQ	96.51 ± 16.95	105.80 ± 14.66	99.67 ± 9.01	95.73 ± 14.05
CSHQ	56.78 ± 6.56^#^	61.78 ± 5.04	54.81 ± 7.61^*^	48.43 ± 11.40

SRS, Social Responsiveness Scales; CEBQ, Children’s Eating Behavior Questionnaire; CSHQ, Children’s Sleep Habits Questionnaire; GI: gastrointestinal symptoms; TD, typical development.

* Significantly different from TD^GI^ vs. TD^NOGI^, *p* < 0.05.

# Significantly different from ASD^GI^ vs. ASD^NOGI^, *p* < 0.05.

### SCFA levels in feces

**Figure 1:**
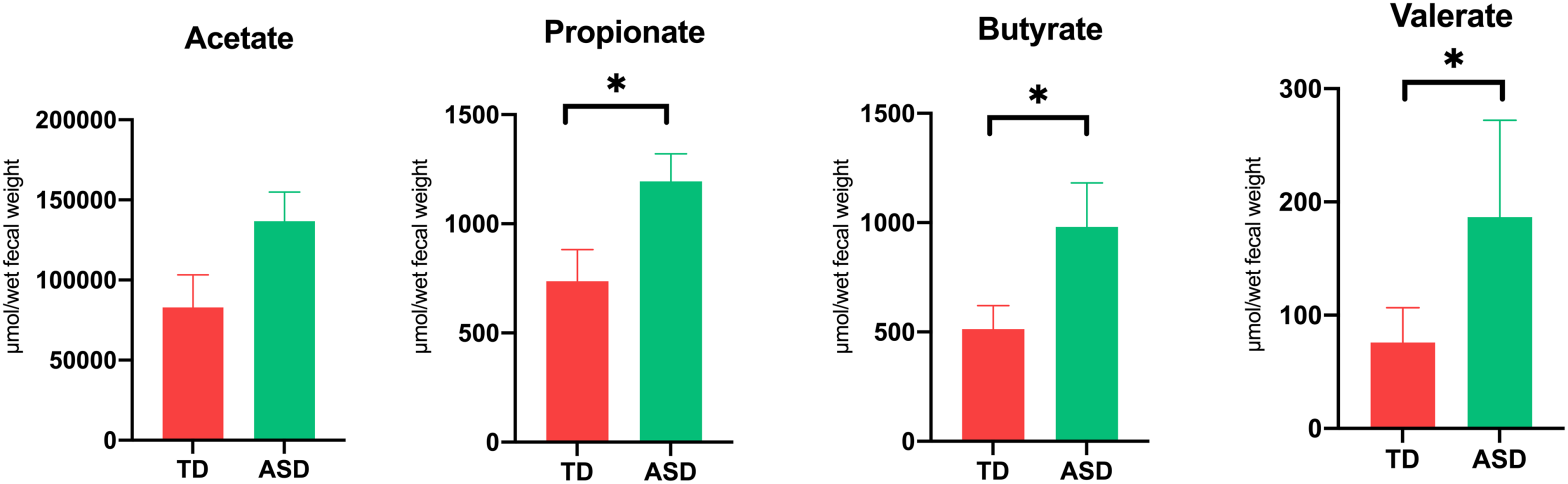
Elevated short-chain fatty acid levels in ASD individuals. The levels of acetic, propionic, butyric, and valeric acid in feces. Each value was presented as the mean ± SEM. ^*^p < 0.05.

We compared the concentrations of SCFAs, including acetic acid (AA), propionic acid (PPA), butyric acid (BTA), and valeric acid (VA), between the groups. The results showed that the PPA (1,195 ± 125.4 vs. 735.6 ± 145.5), BTA (981.1 ± 201.1 vs. 513.5 ± 107.0), and VA (186.6 ± 85.4 vs. 75.9 ± 30.7), levels were significantly higher in the ASD group than in the TD group (Figure 1; *p* < 0.05, Mean ± SEM). We correlated analyzed The Social Responsiveness Scale, The Children’s Sleep Habits Questionnaire, and The Children’s Eating Behavior Questionnaire with short-chain fatty acids. The results show that butyric acid was moderately negatively correlated with SRS (−0.47) and moderately positively correlated (0.47) with CSHQ.

### The distribution of microbiota and associations with SCFAs in the ASD and TD groups

**Figure 2:**
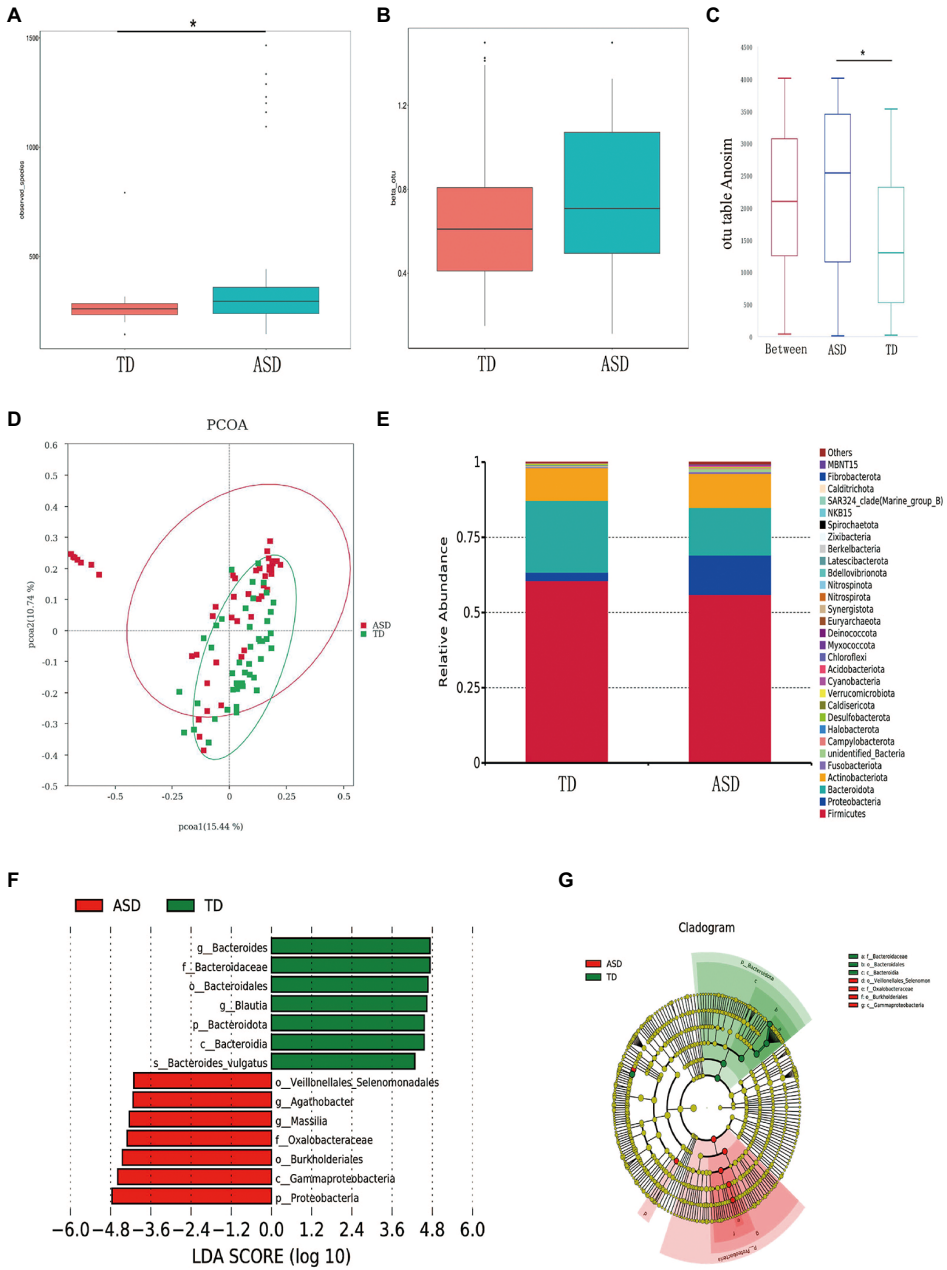
The shape of the microbiota in typically developing (TD) children and children with ASD. **(A)** Comparison of bacterial richness (observed_species) and **(B)** diversity (beta_otu). **(C)** ANOSIM was used to compare within-and between-group similarities (R = 0.1337, *p* = 0.001). **(D)** Principal coordinate analysis (PCoA) of the microbiota in the TD and ASD groups by using unweighted UniFrac distance metrics. **(E)** The relative abundance of different taxa at the phylum level in ASD and TD. **(F)** LDA scores for bacterial taxa that diverge in abundance between TD and ASD (LDA > 4). **(G)** The cladogram illustrates the enriched taxa of gut microbiota in ASD and TD.

A total of 107,378 high-quality sequences were obtained after paired-end read merging and error correction of 16S rRNA sequencing data. On the basis of 97% sequence identity, the amplicons were divided into 3,615 operational taxonomic units. Figure 2 shows that alpha diversity was higher in the ASD group, whereas the two groups showed no significant difference in beta diversity on the basis of Bray–Curtis distances (Figures 2A,B). Analysis of similarities (ANOSIM) indicated that the differences between groups were significantly greater than those within groups (R = 0.1337, *p* = 0.001; Figure 2C). Principal coordinate analysis was used to determine the degree of similarity between the bacterial diversities of the two groups using unweighted UniFrac distance metrics (Figure 2D). The bacterial phyla showing the top 30 relative abundances in each group are shown in Figure 2E. Linear discriminant analysis (LDA > 3.5) was used to produce a dimension reduction to measure the extent of the effects of the different microbiota. The results showed that in comparison with the TD group, Veillonellales_Selenomonadales, *Agathobacter*, *Massilia*, Oxalobacteraceae, Burkholderiales, Gammaproteobacteria, and Proteobacteria were significantly enriched in the ASD group (Figures 2F,G).

**Figure 3:**
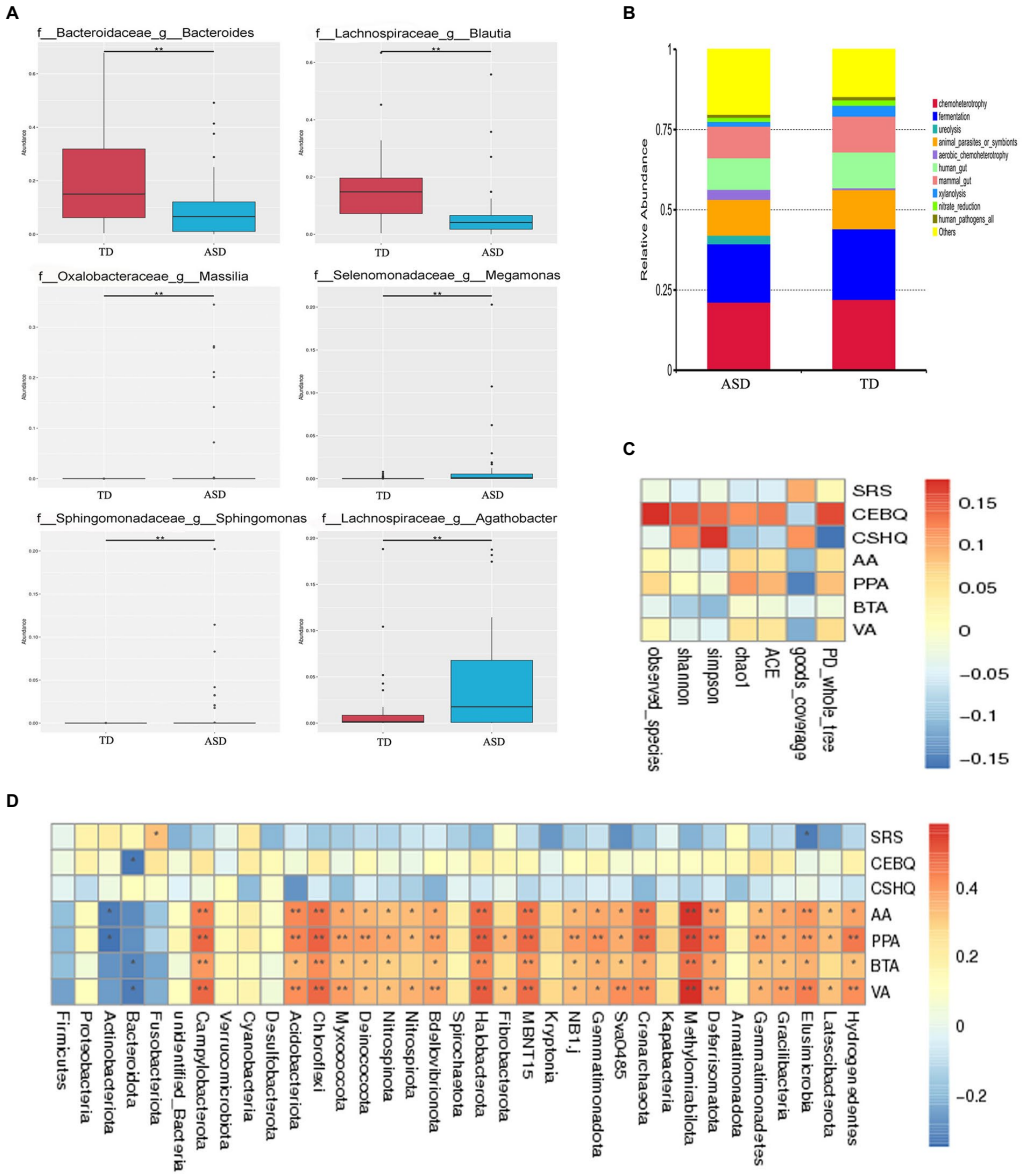
The specific microbial changes between the two groups and their associations with SCFAs. **(A)** The top 6 relative abundance genus levels of gut microbiota between the TD and ASD groups based on metastat analysis. **(B)** The FAPROTAX database was used to predict the functions of bacteria in both groups. **(C)** Spearman correlation analysis of the association between fecal SCFA and sociality, eating, sleep questionnaire scores in alpha diversity, and **(D)** in the phylum level in the ASD group.

Next, to characterize possible clinically important bacteria related to ASD, we used metastat analysis to identify the genera showing the top six relative abundance levels between the gut microbiota of the two groups. The findings revealed that *Massilia*, *Megamonas*, *Sphingomonas*, and *Agathobacter* were more abundant, and *Bacteroidetes* and *Blautia* were less abundant in the ASD group (Figure 3A). Furthermore, the FAPROTAX database was used to predict bacterial function in the ASD and TD groups (Figure 3B). We also aimed to elucidate the association between gut microbiota and fecal SCFAs, sociality, eating, and sleep in individuals with ASD. Spearman correlation analysis showed that alpha diversity was significantly positively correlated with eating behaviors (Figure 3C; Observed_species, ACE, Chao1, Shannon, Simpson, and PD_whole_tree). In addition, we analyzed the relative abundance of the top 35 bacterial phyla and found that Hydrogenedentes, Elusimicrobia, Methylomirabilota, Crenarchaeota, MBNT15, Halobacteria, Chloroflexi, Actinobacteria, and *Campylobacter* were strongly associated with SCFAs (Figure 3D). In contrast, Elusimicrobia was negatively associated with the SRS score, while Bacteridota was significantly negatively correlated with eating habits.

### GI symptoms affect the gut microbiota

**Figure 4:**
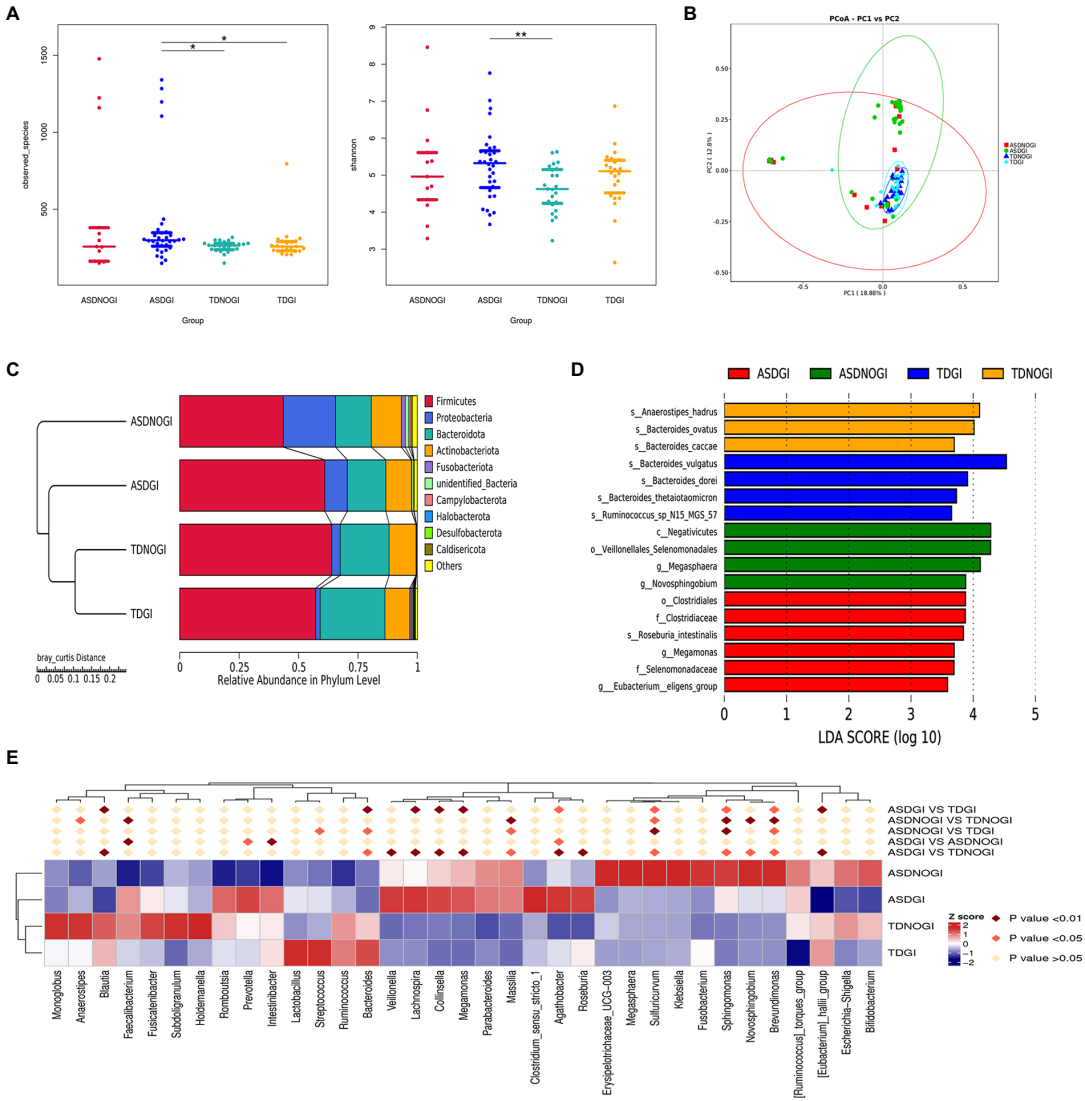
The gastrointestinal symptoms affected the microbiome in the ASD and control groups. **(A)** Evaluated bacterial abundance (observed species) and diversity (beta otu). **(B)** PCA of bacterial beta diversity based on the Bray–Curtis distance. **(C)** The major contributing phyla of the gut microbiota according to the Bray–Curtis distance. **(D)** Distribution histogram of phylum level based on linear discriminant analysis (LDA > 3.5). **(E)** The heatmap of the top 35 phyla in different groups.

A growing number of studies have demonstrated that over 50% of children with ASD report comorbid GI disturbances. We further investigated whether GI symptoms have the potential to reprogram the gut microbiome of individuals with ASD. As shown in Figure 4A, the ASD plus GI symptoms group showed greater richness and diversity than the TD with no GI symptoms group (Observed_species, Shannon; Figure 4A). We used principal component analysis based on the unweighted UniFrac distance to show that the bacterial community composition differed among the four groups (Figure 4B). The phyla showing the top 10 relative abundance values in the different groups are shown in Figure 4C. LDA Effect Size analysis (LDA threshold of 3.5) showed that Clostridiales, Clostridiaceae, *Roseburia intestinalis*, *Megamonas*, Selenomonadaceae, and *Eubacterium eligens* groups were significantly enriched in the ASD with GI symptoms group (Figure 4D). At the phylum level, the top 35 microbiota are shown in Figure 4E.

### ASD with GI symptoms vs. TD with no GI symptoms

**Figure 5:**
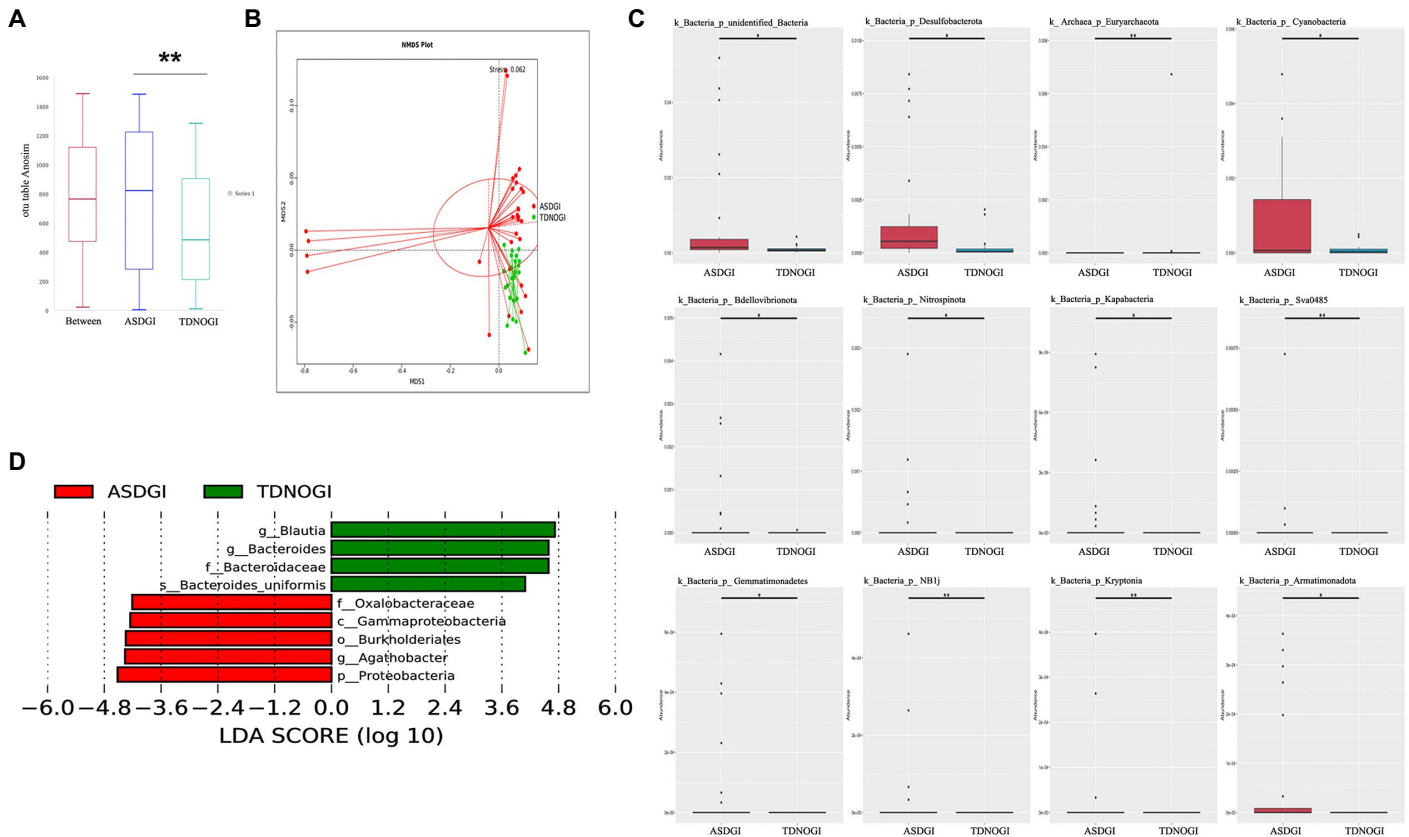
The shape of the microbiota with and without GI symptoms. **(A)** ANOSIM was used to compare within- and between-group similarities (*p* = 0.005). **(B)** Non-metric multidimensional scaling (NMDS) reflected the dramatic variation between the two groups (stress = 0.062). **(C)** The top 12 relative abundances of the most differentially abundant gut bacterial phyla between ASDGI and TDNOGI. **(D)** Distribution histogram of phylum level based on linear discriminant analysis (LDA = 4). **p = 0.01.

Next, to better understand how GI symptoms potentially interact with the gut microbiota, we selected the ASD with GI symptoms and TD with no GI symptoms groups for comparison. As shown in Figure 5A, ANOSIM analyses showed significant differences between groups (*p* = 0.005). Non-metric multidimensional scaling reflected the dramatic variation between the two groups (Figure 5B; stress = 0.062). In addition, we identified the top 12 differentially abundant taxa based on metastat analysis (Figure 5C), and we further dissected several of the relatively abundant microbiota in the ASD with GI symptoms group, including Oxalobacteraceae, Gamaproteobacteria, Burkholderiates, *Agathobacter*, and Proteobacteria (Figure 5D; LDA = 4). The microbiota differences between the ASD patients with and without GI symptoms are presented in Supplementary Figure S1.

## Discussion

In this study, we evaluated the associations between the core co-occurring conditions, SCFA, and gut microbiota in individuals with ASD with or without GI issues. And consistent with the findings of other studies, children with ASD had a high rate of gastrointestinal problems (McElhanon et al., 2014; Wasilewska and Klukowski, 2015; Leader et al., 2022a,b). In addition, children with ASD displayed more severe social impairment, sleep issues, fussy eating, and a desire for drink than children with TD. However, gastrointestinal symptoms had no functional impact on the social, eating, or sleeping patterns of autistic children. And fecal SCFA content significantly increased in ASD group. We further discovered that the ASD, TD, and GI subgroups had distinct microbiome profiles. Surprisingly, the ASD group demonstrated greater richness, and those who also experienced GI symptoms exhibited notable richness and diversity.

Our findings matched the previous findings showing that 78% of individuals with ASD had gastrointestinal symptoms, and the most common GI symptoms were smelly stools and constipation. In a recent review, Radu Lefter et al. found that 83% of the studies in the literature reported GI symptoms in children with ASD (Lefter et al., 2019), and the most common type of GI disorder was functional constipation, which was observed in 85% of the individuals with ASD. Additionally, individuals with ASD and co-existent GI diseases had less expressive language and more social impairment. The authors claimed that GI disorders are not caused by diet or eating habits (Gorrindo et al., 2012). Other studies have found that children with GI issues have more severe ASD core symptoms (social interaction, social communication, self-injurious activity, and sleep problems) than those without GI problems, indicating that GI disorders may exacerbate or even partially cause behavioral issues in children with ASD (McElhanon et al., 2014; Babinska et al., 2020; Settanni et al., 2021). In line with the report by Xiao-Lei Yang, GI symptoms in patients with ASD were associated with more severe ASD core symptoms, and ASD with GI symptoms may be a clinical subtype of ASD (Yang X L et al., 2018). However, our results showed that the core symptom of autism, social impairment, did not show a significant differee with or without GI symptoms. The debate over whether GI disturbances appear in combination with autism symptoms or are influenced by the microbiota remains controversial.

We further investigated sleep disorders and eating patterns, which are the most common co-occurring symptoms of autism and are associated with GI disorders. A number of studies have indicated that individuals with ASD have different sleep and dietary patterns in comparison with TD individuals, and the severity of autistic symptoms and sleep problems have a statistically significant relationship (Cohen et al., 2014). Our findings indicate that GI symptoms only affected the TD group, and the ASD without GI group had higher CSHQ scores than the ASD plus GI group.

According to previous studies, children with ASD are at an increased risk of sleep disturbances, with rates ranging from 60% to 96%, and young children experience more sleep problems (Samanta et al., 2020). The most prevalent sleep problems are sleep onset difficulties, longer sleep latency, earlier wake-up time, and more night awakenings (Krakowiak et al., 2008; Richdale and Schreck, 2009). Other studies have shown that insufficient sleep exacerbates the core ASD symptoms (e.g., repetitive behaviors and social and communication difficulties), and that children with insufficient sleep show more internalizing and externalizing behavior problems and poorer adaptive skill development than children with ASD without sleep problems (Goldman et al., 2009; Sikora et al., 2012; Fadini et al., 2015). Along with increased sleep impairments, ASD symptoms are associated with significantly higher rates of GI symptoms (Leader et al., 2022a,b). GI symptoms also predicted sleep issues through the CSHQ total score (Hollway et al., 2013). Vissoker et al. reported that GI dysfunction frequently indicates the onset of sleep issues, eating issues, challenging behaviors, and compulsive behaviors (Hodge et al., 2014; Vissoker et al., 2015). The potential reasons for these inconsistent results may be that sleep symptoms are one of the characteristics of ASD, individuals are relatively young, and gastrointestinal symptoms have a limited effect on sleep.

Regarding eating patterns, although the two groups showed no significant differences in the scores for the CEBQ, the ASD group showed more food fussiness and desire to drink. This is in accordance with previous findings showing that eating and feeding difficulties, most often food selectivity or picky eating (nearly 70%), are more prevalent in children with ASD (Baraskewich et al., 2021). Moreover, nutritional challenges affect up to 79% of children with ASD (Malhi et al., 2017). A growing body of research has established links between eating problems, GI disorders, and microbiota in children with ASD. However, previous research on this subject has yielded conflicting results. Twachtman-Reilly et al. found that restricted and repetitive behaviors, including hypo-or hyperreactivity and sensory processing difficulties, may manifest as food selectivity (Twachtman-Reilly et al., 2008). However, for many children with ASD, eating and feeding problems may originate from the underlying gastrointestinal dysfunction. Children who complained of stomach pain appeared to have bloated stomachs and gagged more frequently during meals. A greater frequency of gas passage was associated with a less irrational dislike of specific foods. Kang et al. reported that GI symptoms were associated with significantly higher rates of food intolerance (Kang et al., 2014). The authors postulated that food intolerance could lead to mucosal inflammation and cause symptoms of GI disorders such as reflux, gas and bloating, diarrhea, or constipation. Interestingly, the latest research indicates that autistic characteristics, such as restricted interests, are associated with a diet that is less diverse (Yap et al., 2021). Thus, based on previous studies and our results, we hypothesized that although GI symptoms dramatically increase in ASD, they do not affect the core symptoms of autism. GI symptoms may be affected by the gut microbiota and its metabolism.

We hypothesized that GI disorders may display a different pattern in gut microbiota. First, we compared the gut microbiota of individuals with ASD and TD individuals. Surprisingly, when compared to TD individuals, individuals with ASD showed lower α-diversity and alteration of gut microbiota, which is consistent with the results of previous studies (Finegold et al., 2010; De Angelis et al., 2013; Cao et al., 2021). In contrast, Ma et al. found decreased gut microbial diversity in preschool children with ASD (Ma et al., 2019). In addition, Zhou Dan et al. observed that individuals with ASD and constipation showed increased α-diversity than indiviuals with ASD without constipation, and the α-diversity of children with ASD did not change with age, whereas that of TD children increased with age (Dan et al., 2020). These results indicate that in young children with ASD, the microbiota is in the dynastic developmental and robust stage, which may not be “worse” (or showing less diversity) than that in TD children.

However, we found significant differences in the microbial composition of the two groups. In the ASD group, Veillonellales_Selenomonadales, *Agathobacter*, *Massilia*, Oxalobacteraceae, Burkholderiales, Gammaproteobacteria, *Megamonas*, *Sphingomonas*, and Proteobacteria were significantly enriched, whereas Bacteroidetes and *Blautia* were less enriched. Furthermore, significantly higher levels of the microbiota in the ASD group were also related to GI disorders. Although gut microbiota dysbiosis in ASD has been described in many published studies, there remains a lack of consensus on the exact composition of the microbiota, and the more consistent results are that children with autism have a lower prevalence of Bifidobacterium and Firmicutes and a higher prevalence of Clostridium, Bacteroidetes, Desulfovibrio, and Lactobacillus in their gut microbiota when compared to the control group (Li et al., 2017; Yang Y et al., 2018; Bojović et al., 2020).

Among the microbiota identified in our ASD group, some were closely related to human pathophysiology. For example, Veillonellales, a pro-inflammatory and lactate-fermenting bacterium, was found to be more prevalent in patients with irritable bowel syndrome, who can experience GI symptoms (Herndon et al., 2020). Selenomonadales and *Sphingomonas* were found to be significantly decreased in the ASD group (Lee et al., 2017; Sun et al., 2019). Burkholderiales, *Megamonas*, and Gammaproteobacteria are increased in the ASD group (Li et al., 2019; Liu et al., 2019). Interestingly, Hua et al. revealed that *Agathobacter* may affect the severity of sleep disorders and autism core symptoms in children with ASD (Hua et al., 2020). In addition, the relative abundance of Proteobacteria was evaluated in 11 trials and a moderate but significant effect was found (Iglesias-Vázquez et al., 2020). The reasons for these inconsistent results may include dynamic microbiota development, disease heterogeneity, diet, age, and differences in the detection methods.

Next, in analyses of the relationship between GI symptoms and microbiota, the cause and effect of GI dysfunction in children with ASD are not fully understood, but the gut microbiota appears to play a significant role in this process. For example, probiotic treatment with *L. reuteri* improved ASD behavior in both animal and human studies, suggesting a potential therapeutic option for GI symptoms and ASD behaviors (Hsiao et al., 2013; Sgritta et al., 2019). Additionally, rather than GI symptoms or diet, gut microbiota changes have been reported to be linked to behavioral symptoms (Peretti et al., 2019). According to Strati et al., constipation is associated with a variety of bacterial taxa, depending on whether an individual has ASD (Strati et al., 2017). Given the link between a poor diet and gut microbiome biomarker abundance, poor diets can conceivably cause gut dysbiosis (Liu et al., 2016).

Since the level of SCFAs was significantly higher in patients with autism in the current study, we hypothesized that SFCAs are associated with GI symptoms. A growing body of research indicates that SCFAs, the metabolic products of the gut microbiota, are linked to autism and have the potential to show widespread effects on GI function, the brain, and behavior (Macfabe, 2012). We observed that the phylum levels of Hydrogenedentes, Elusimicrobia, Methylomirabilota, Crenarchaeota, MBNT15, Halobacteria, Chloroflexi, Actinobacteria, and *Campylobacter* were strongly associated with SCFAs. In addition, 74 gut bacteria produce SCFA, most of which are from the probiotic genera *Lactobacillus*, *Bifidobacterium*, and *Clostridium*. *Clostridium* and *Bifidobacterium* are the primary bacteria that produce acetate, and *Clostridium* also produces butyrate and propionate (Zhu et al., 2022). There was no significant difference between the ASD and TD groups (Supplementary Table S3). Interestingly, Clostridiales, Clostridiaceae, *Roseburia intestinalis*, *Megamonas*, and *Eubacterium eligens* (Figure 4D), which are the dominant SCFA-producing bacteria according to previous reports (Kasahara et al., 2018; Mukherjee et al., 2020), were significantly enriched in the ASD group with GI symptoms. For example, butyrate produced by *Roseburia* and *Megamonas* can ferment glucose into acetate and propionate (Chevrot et al., 2008; Sakon et al., 2008).

Previous research on fecal SCFA in ASD has been inconsistent, especially involving GI disturbances and microbiota. Wang et al. noted that fecal levels of acetic, butyric, isobutyric, valeric, and isovaleric acids were significantly higher in children with ASD (Wang et al., 2012). A recent investigation of fecal SCFA in constipated ASD children indicated a two-fold increase in VA concentrations associated with Acidobacteria in children with ASD compared to TD children (Hua et al., 2020). However, SCFA was not different in adults with ASD (Gonzales et al., 2021), and De Angelis et al. reported that total short-and medium-chain fatty acids were significantly higher in TD individuals than in ASD individuals (De Angelis et al., 2013). Individuals with Rett syndrome (the leading monogenic cause of ASD) had less bacterial gut microbiota richness and diversity than TD individuals and displayed high levels of SCFAs, especially isovalerate and isobutyrate propionate. Additionally, the gut microbiota is not dependent on constipation status, and this dysbiotic microbiota produces altered SCFA profiles (Strati et al., 2016). Adams et al. reported a strong link between GI symptoms and the severity of autism, and the total amount of SCFA (acetate, propionate, and valerate) was significantly lower in individuals with ASD in their study, which appears to be partly due to probiotic usage (Adams et al., 2011). GI symptoms, including constipation, may be exacerbated by abnormally high levels of SCFA in the intestines (Barcelo et al., 2000). This inconsistent outcome could be attributed to the interaction of gastrointestinal symptoms, age, diet, testing time, and methods.

Enteric SCFAs have a variety of mechanisms of action in autism, not only by strongly influencing tight junctions and production of mucin in the gut, but also through their effects on gene expression, brain function, and behavior in rat and *in vitro* ASD cell models (Nankova et al., 2014; MacFabe, 2015). For example, AA is the most abundant SCFA and is involved in the maintenance of normal intestinal structure, integrity, and function (Parada Venegas et al., 2019). Butyrate has been shown to reduce inflammation, improve epithelial barrier function, and protect against enteric pathogen colonization and infection (Liu et al., 2018, 2019). These results suggest that high SCFA levels may affect GI symptoms in ASD individuals.

### Study limitations

Despite its novelty, our study has some limitations. First, this was not a longitudinal study, and the participants were preschoolers. Second, gold-standard diagnostic scales, such as the Autism Diagnostic Observation Schedule and the Autism Diagnostic Interview—Revised, are difficult to obtain in the study country. Furthermore, the microbiota was captured by 16S rRNA and not by metagenomics. Since ASD is a highly heterogeneous disorder, it is more reasonable to perform a subgroup analysis to assess factors such as severity.

### Conclusion

Based on the results of this study, we further confirmed that GI disturbances are widespread in autism and that the presence of gastrointestinal symptoms has no noticeable impact on autism core symptoms. However, the ASD microbiota structure appears to differ in the presence of GI symptoms, and elevated SCFA levels may be associated with GI symptoms and microbiota. We hypothesize that the microbiota is robustly developing, and the whole profile of microbiota and functions may not be very different from TD children, but changes in metabolism may affect the GI symptoms of autism. Due to the heterogeneity of autism, it is logical to stratify future studies based on the severity of ASD, GI symptoms, and age distribution.

## Data availability statement

The datasets presented in this study can be found in online repositories. The names of the repository/repositories and accession number(s) can be found in the article/Supplementary material.

## Ethics statement

The studies involving human participants were reviewed and approved by the Southern Medical University Ethics Committee. Written informed consent to participate in this study was provided by the participants’ legal guardian/next of kin.

## Author contributions

WD and SW designed the study, performed analyses, and drafted this manuscript. SW, FW, YX, HK, HH, and Li helped recruit children and performed a systematic literature search. XX and WD interpreted the data. WD, SW, FL, FW, YX, YLi, YLv, HK, ZL, PL, HH, YC, and XX critically reviewed the manuscript for intellectual content, approved the final version, and have access to data. WD, SW, FL, FW, YX, YLi, YLv, and YC are co-first authors. All authors contributed to the article and approved the submitted version.

## Funding

This study was supported by the Administration of Traditional Chinese Medicine of Guangdong Province, China (20221091), the National Natural Science Foundation of China (grant numbers 81770529 and 82100609), Science and Technology Foundation of Guangzhou, China (202103000071), Hainan Provincial Natural Science Foundation of China (no. 821QN0982), and Guangdong Gastrointestinal Disease Research Center (grant number 2017B02029003), and Southern Hospital of Southern Medical University, Dean’s Fund (2019B036).

## Conflict of interest

The authors declare that the research was conducted in the absence of any commercial or financial relationships that could be construed as a potential conflict of interest.

## Publisher’s note

All claims expressed in this article are solely those of the authors and do not necessarily represent those of their affiliated organizations, or those of the publisher, the editors and the reviewers. Any product that may be evaluated in this article, or claim that may be made by its manufacturer, is not guaranteed or endorsed by the publisher.

## Supplementary material

SUPPLEMENTARY FIGURE S1 Gastrointestinal symptoms affected the microbiome in ASD.

SUPPLEMENTARY FILE 1 16S Analysis methods.
