# Supplementary material for: Gastrointestinal symptoms have a minor impact on autism spectrum disorder and associations with gut microbiota and short-chain fatty acids
Source: Front Microbiol. 2022 Oct 7;13:1000419. doi: 10.3389/fmicb.2022.1000419 (PMC9585932; doi:10.3389/fmicb.2022.1000419)
Supplement: SUPPLEMENTARY FIGURE S1 — Gastrointestinal symptoms affected the microbiome in ASD. [file Data_Sheet_1.zip › Methods.doc]

Methods


Sequencing

1.	Extraction of genome DNA

Total genome DNA from samples was extracted using CTAB/SDS method. DNA concentration and purity were monitored on 1% agarose gels. According to the concentration, DNA was diluted to 1ng/¦ÌL using sterile water.

2.	Amplicon Generation

16S rRNA/18SrRNA/ITS genes of distinct Regions (16SV4/16SV3/16SV3-V4/16SV4-V5, 18S V4/18S V9, ITS1/ITS2, Arc V4) were amplified used specific primer (e.g. 16S V4: 515F-806R, 18S V4: 528F-706R, 18S V9: 1380F-1510R, et. al) with the barcode. All PCR reactions were carried out with Phusion® High-Fidelity PCR Master Mix (New England Biolabs).

3.	PCR Products quantification and qualification

Mix same volume of 1X loading buffer (contained SYB green) with PCR products and operate electrophoresis on 2% agarose gel for detection. Samples with bright main strip between 400-450bp were chosen for further experiments.

4.	PCR Products Mixing and Purification

PCR products was mixed in equidensity ratios. Then, mixture PCR products was purified with Qiagen Gel Extraction Kit (Qiagen, Germany).

5.	Library preparation and sequencing

Sequencing libraries were generated usingTruSeq® DNA PCR-Free Sample Preparation Kit (Illumina, USA) following manufacturer's recommendations and index codes were added. The library quality was assessed on the Qubit@ 2.0 Fluorometer (Thermo Scientific) and Agilent Bioanalyzer 2100 system. At last, the library was sequenced on an IlluminaHiSeq2500 platform and 250 bp paired-end reads were generated.


Data analysis

1.	Paired-end reads assembly and quality control

1.1	Data split
Paired-end reads was assigned to samples based on their unique barcode and truncated by cutting off the barcode and primer sequence.

1.2	Sequence assembly
Paired-end reads were merged using FLASH (V1.2.7,http://ccb.jhu.edu/software/FLASH/) [1],a very fast and accurate analysis tool, which was designed to merge paired-end reads when at least some of the reads overlap the read generated from the opposite end of the same DNA fragment, and the splicing sequences were called raw tags.

1.3	Data Filtration
Quality filtering on the raw tags were performed under specific filtering conditions to obtain the high-quality clean tags[2]according to the QIIME(V1.7.0, http://qiime.org/index.html)[3]quality controlled process.

1.4	Chimera removal
The tags were compared with the reference database(Gold database, http://drive5.com/uchime/uchime_download.html)using UCHIME algorithm(UCHIME Algorithm, http://www.drive5.com/usearch/manual/uchime_algo.html)[4]to detect chimera sequences, and then the chimera sequences were removed[5]. Then the Effective Tags finally obtained.

2.	OTU cluster and Species annotation

2.1	OTU Production
Sequences analysis were performed by Uparse software (Uparse v7.0.1001£¬ http://drive5.com/uparse/)[6]. Sequences with ¡Ý97% similarity were assigned to the same OTUs. Representative sequence for each OTU was screened for further annotation.

2.2	Species annotation
For each representative sequence, the GreenGene Database (http://greengenes.lbl.gov/cgi-bin/nph-index.cgi)[7]was used based on RDP

Classifier (Version 2.2, http://sourceforge.net/projects/rdp-classifier/)[8]algorithmto annotate taxonomic information.

2.3	Phylogenetic relationship Construction
In order to study phylogenetic relationship of different OTUs, and the difference of the dominant species in different samples(groups), multiple sequence alignment were conducted using the MUSCLE software (Version 3.8.31£¬ http://www.drive5.com/muscle/)[9].

2.4	Data Normalization
OTUs abundance information were normalized using a standard of sequence number corresponding to the sample with the least sequences. Subsequent analysis of alpha diversity and beta diversity were all performed basing on this output normalized data.

3.	Alpha Diversity
Alpha diversity is applied in analyzing complexity of species diversity for a sample through6 indices, including Observed-species, Chao1, Shannon, Simpson, ACE, Good-coverage. All these indices in our samples were calculated with QIIME (Version 1.7.0) and displayed with R software (Version 2.15.3).

Two indices were selected to identify Community richness: Chao - the Chao1 estimator (http://www.mothur.org/wiki/Chao);
ACE - the ACE estimator (http://www.mothur.org/wiki/Ace);

Two indices were used to identify Community diversity:

Shannon - the Shannon index (http://www.mothur.org/wiki/Shannon);

Simpson - the Simpson index (http://www.mothur.org/wiki/Simpson);

One indice to characterized Sequencing depth:

Coverage - the Good's coverage (http://www.mothur.org/wiki/Coverage)

4.	Beta Diversity

Beta diversity analysis was used to evaluate differences of samples in species complexity, Beta diversity on both weighted and unweighted unifrac were calculated by QIIME software (Version 1.7.0).
Cluster analysis was preceded by principal component analysis (PCA), which was applied to reduce the dimension of the original variables using the FactoMineR package and ggplot2 package in R software (Version 2.15.3).

Principal Coordinate Analysis (PCoA) was performed to get principal coordinates and visualize from complex, multidimensional data. A distance matrix of weighted or unweighted unifrac among samples obtained before was transformed to a new set of orthogonal axes, by which the maximum variation factor is demonstrated by first principal coordinate, and the second maximum one by the second principal coordinate, and so on. PCoA analysis was displayed by WGCNA package, stat packages and ggplot2 package in R software (Version 2.15.3).

Unweighted Pair-group Method with Arithmetic Means (UPGMA) Clustering was performed as a type of hierarchical clustering method to interpret the distance matrix using average linkage and was conducted by QIIME software (Version 1.7.0).


Reference

[1]	Magoč T, Salzberg S L. FLASH: fast length adjustment of short reads to improve genome assemblies. Bioinformatics 27.21 (2011): 2957-2963.

[2]	Bokulich, Nicholas A., et al. Quality-filtering vastly improves diversity estimates from Illuminaamplicon sequencing. Nature methods 10.1 (2013): 57-59.

[3]	Caporaso, J. Gregory, et al. QIIME allows analysis of high-throughput community sequencing data. Nature methods 7.5 (2010): 335-336.

[4]	Edgar, Robert C., et al. UCHIME improves sensitivity and speed of chimera detection. Bioinformatics 27.16 (2011): 2194-2200.

[5]	Haas, Brian J., et al. Chimeric 16S rRNA sequence formation and detection in Sanger and 454-pyrosequenced PCR amplicons.Genome research 21.3 (2011): 494-504.

[6]	Edgar, Robert C. UPARSE: highly accurate OTU sequences from microbial amplicon reads. Nature methods 10.10 (2013): 996-998.

[7]	DeSantis, Todd Z., et al. Greengenes, a chimera-checked 16S rRNA gene database and workbench compatible with ARB. Applied and environmental microbiology 72.7 (2006): 5069-5072.

[8]	Wang, Qiong, et al. Naive Bayesian classifier for rapid assignment of rRNA sequences into the new bacterial taxonomy. Applied and environmental microbiology 73.16 (2007): 5261-5267.

[9]	Edgar R C. MUSCLE: multiple sequence alignment with high accuracy and high throughput. Nucleic acids research32.5(2004): 1792-1797.
