# Supplementary material for: Gastrointestinal symptoms have a minor impact on autism spectrum disorder and associations with gut microbiota and short-chain fatty acids
Source: Front Microbiol. 2022 Oct 7;13:1000419. doi: 10.3389/fmicb.2022.1000419 (PMC9585932; doi:10.3389/fmicb.2022.1000419)
Supplement: SUPPLEMENTARY FIGURE S1 — Gastrointestinal symptoms affected the microbiome in ASD. [file Data_Sheet_1.zip › Table S1.docx]

| **Table S1.** Values of the Children Sleep Habits Questionnaire. | | | |
| --- | --- | --- | --- |
|  | ASD (n=45) | TD (n=45) | P |
| Total sleep time | 8.76 ± 1.61 | 9.21 ± 0.62 | 0.0894 |
| Bedtime resistance * | 12.29 ± 2.41 | 10.75 ± 2.57 | 0.0045 |
| Sleep anxiety | 7.71 ± 2.10 | 7.23 ± 2.13 | 0.2822 |
| Sleep duration | 5.60 ± 1.71 | 4.73 ± 1.80 | 0.0212 |
| Sleep-disordered breathing | 3.40 ± 0.65 | 3.23 ± 0.83 | 0.2784 |
| Parasomnias * | 9.38 ± 1.71 | 8.37 ± 2.27 | 0.0208 |
| Daytime sleepiness * | 13.38 ± 2.58 | 12.14 ± 3.10 | 0.0428 |
| Night waking | 3.67 ± 1.30 | 3.30 ± 0.90 | 0.1217 |
| Sleep onset delay * | 2.18 ± 0.72 | 1.72 ± 0.76 | 0.005 |
| *p < 0.05 |  |  |  |
