# Supplementary material for: Gastrointestinal symptoms have a minor impact on autism spectrum disorder and associations with gut microbiota and short-chain fatty acids
Source: Front Microbiol. 2022 Oct 7;13:1000419. doi: 10.3389/fmicb.2022.1000419 (PMC9585932; doi:10.3389/fmicb.2022.1000419)
Supplement: SUPPLEMENTARY FIGURE S1 — Gastrointestinal symptoms affected the microbiome in ASD. [file Data_Sheet_1.zip › Table S2.docx]

| **Table S2.** Values of the Children’s Eating Behavior Questionnaire. | | | |
| --- | --- | --- | --- |
|  | ASD (n=45) | TD (n=45) | P |
| Emotional overeating | 9.40 ± 2.10 | 10.89 ± 5.16 | 0.0777 |
| Enjoyment of food | 12.51 ± 2.93 | 12.34 ± 2.12 | 0.7548 |
| Food responsiveness | 13.96 ± 5.01 | 13.09 ± 4.66 | 0.4015 |
| Desire to drink * | 8.47 ± 3.45 | 6.80 ± 2.58 | 0.0115 |
| Emotional undereating | 11.18 ± 3.07 | 11.50 ± 2.13 | 0.5673 |
| Satiety responsiveness | 14.40 ± 2.62 | 13.68 ± 2.04 | 0.153 |
| Food fussiness * | 19.42 ± 4.85 | 17.05 ± 4.10 | 0.0146 |
| Slowness in eating | 11.31 ± 3.72 | 12.32 ± 3.14 | 0.1716 |
| *p < 0.05 |  |  |  |
