# Supplementary figures and images for: Gastrointestinal symptoms have a minor impact on autism spectrum disorder and associations with gut microbiota and short-chain fatty acids
Source: Front Microbiol. 2022 Oct 7;13:1000419. doi: 10.3389/fmicb.2022.1000419 (PMC9585932; doi:10.3389/fmicb.2022.1000419)

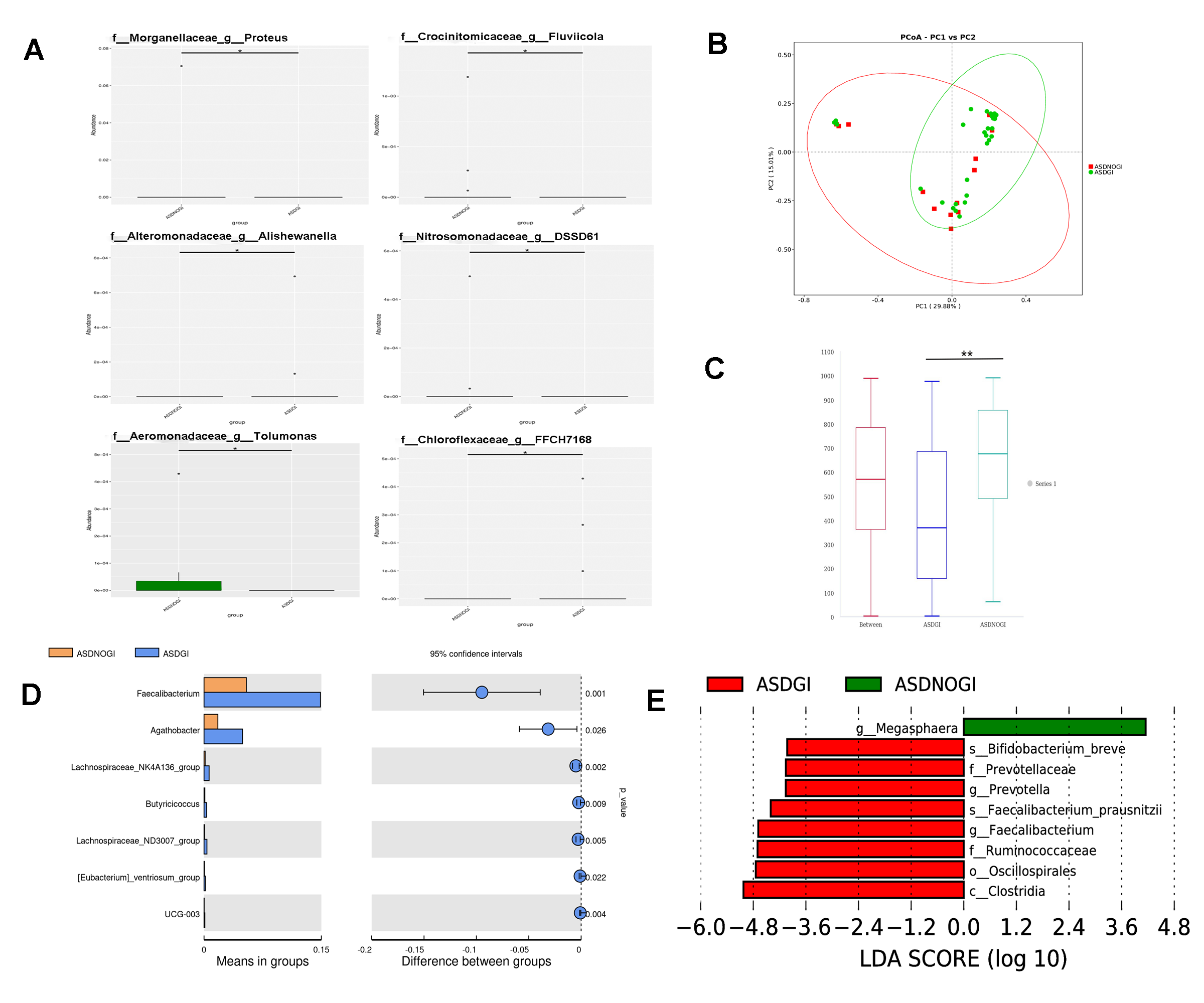

Supplement: SUPPLEMENTARY FIGURE S1 — Gastrointestinal symptoms affected the microbiome in ASD. [file Data_Sheet_1.zip › Supplementary Image 1.TIF]
